# The depressor response to intracerebroventricular hypotonic saline is sensitive to TRPV4 antagonist RN1734

**DOI:** 10.3389/fphar.2015.00083

**Published:** 2015-04-23

**Authors:** Claire H. Feetham, Nicolas Nunn, Richard Barrett-Jolley

**Affiliations:** Department of Musculoskeletal Biology, Institute of Ageing and Chronic Disease, University of LiverpoolLiverpool, UK

**Keywords:** ion channel, TRPV4, blood pressure, heart rate, osmolality, cell volume

## Abstract

Several reports have shown that the periventricular region of the brain, including the paraventricular nucleus (PVN), is critical to sensing and responding to changes in plasma osmolality. Further studies also implicate the transient receptor potential ion channel, type V4 (TRPV4) channel in this homeostatic behavior. In previous work we have shown that TRPV4 ion channels couple to calcium-activated potassium channels in the PVN to decrease action potential firing frequency in response to hypotonicity. In the present study we investigated whether, similarly, intracerebroventricular (ICV) application of hypotonic solutions modulated cardiovascular parameters, and if so whether this was sensitive to a TRPV4 channel inhibitor. We found that ICV injection of 270 mOsmol artificial cerebrospinal fluid (ACSF) decreased mean blood pressure, but not heart rate, compared to naïve mice or mice injected with 300 mOsmol ACSF. This effect was abolished by treatment with the TRPV4 inhibitor RN1734. These data suggest that periventricular targets within the brain are capable of generating depressor action in response to TRPV4 ion channel activation. Potentially, in the future, the TRPV4 channel, or the TRPV4–K_Ca_ coupling mechanism, may serve as a therapeutic target for treatment of cardiovascular disease.

## Introduction

Body fluid osmolality is usually regulated within an extremely narrow range (∼290–300 mOsmol; Bourque, 2008). This is largely maintained through regulation of renal function, but control areas exist within the central nervous system (CNS). One important reason why animals have evolved to control osmolarity within the CNS is that osmoregulation is a complex process and needs to be integrated with other homeostatic elements. Body systems defend electrolyte composition and osmolarity in parallel with blood pressure (BP) and blood volume (Share and Claybaugh, 1972). In laboratory experiments each of these may be differentially regulated, but within a healthy animal each must be controlled as part of an overall pattern of homeostasis. Within the brain, the area of the hypothalamus surrounding the third ventricle is particularly important for osmoregulation. Key areas identified to date include the SFO, organum vasculosum lamina terminalis (OVLT), circumventricular organs (CVO), medial preoptic area (MPO), and PVN of the hypothalamus (Toney et al., 2003; Stocker et al., 2007). Our particular focus has been on the PVN since this is also an established autonomic control center exerting influence over heart rate (HR) and BP in response to a number of homeostatic challenges including temperature (Cham and Badoer, 2008), day–night cycle (Feetham and Barrett-Jolley, 2014), volume load (Lovick et al., 1993), and osmolarity (Stocker et al., 2004a).

The PVN is conveniently subdivided into two major areas; the parvocellular and magnocellular “sub-nuclei” (Swanson and Sawchenko, 1983). The magnocellular region is a logical site of osmosensation since it contains a high density of neurons that secrete vasopressin (also known as anti-diuretic hormone, ADH) from the neurohypophysis (posterior pituitary; Swanson and Sawchenko, 1983). In addition to its anti-diuretic properties, vasopressin also exerts profound effects on vascular contractility (Share, 1988). The parvocellular region of the PVN is named after the smaller “parvocellular” neurons within. These neurons sub-serve diverse functions. Many release corticotropin-releasing factor (CRF) into the hypophyseal portal circulation, which, in turn, evokes release of adrenocorticotropic hormone (ACTH) from the adenohypophysis (anterior pituitary) and is a key part of the ACTH-adrenal-cortisol axis (Antoni, 1993). Additionally, the parvocellular region of the PVN also contains a number of neurons which modulate autonomic control. These neurons project to areas such as the intermediolateral nucleus (IML) of the spinal cord and synapse with preganglionic sympathetic neurons (Motawei et al., 1999; Barrett-Jolley et al., 2000; Pyner and Coote, 2000). When activated, these “pre-autonomic” neurons elevate HR, BP, and sympathetic nervous activity (SNA) including rSNA (Womack et al., 2007). Some authors have alternatively concluded that the spinally projecting pre-autonomic PVN neurons are neither parvocellular, nor magnocellular, but a family of intermediately sized neurons they named mediocellular neurons (Kiss et al., 1991). Under resting conditions the pre-autonomic parvocellular neurons richly express GABA_A_ receptors (Zaki and Barrett-Jolley, 2002) and exist under a state of tonic inhibition by GABAergic input (Nunn et al., 2011). Furthermore, application of the GABA_A_ antagonist bicuculline evokes increases in rSNA, HR, and BP (Chen et al., 2003). This tonic inhibition is not absolute, since paraventricular application of the GABA_A_ agonist muscamol produces powerful inhibition of SNA with associated decreases of HR and BP (Zhang et al., 2002). Toney et al. (2003), however, report that this response is more apparent in chronically dehydrated rats, where tonic inhibition of the PVN is reduced (Stocker et al., 2004b; Holbein et al., 2014). This reduction in tonic inhibition is due to an additional excitatory input from the MPO, rather than a demonstrable alleviation of the tonic GABAergic inhibition (Stocker and Toney, 2005). Thus, dehydration and hypertonicity applied by either direct application to the hypothalamus or via intra-carotid cannulae, lead to; (i) elevated c-fos expression in pre-autonomic PVN neurons (Stocker et al., 2004a; Gottlieb et al., 2006; Arnhold et al., 2007), (ii) increased spiking activity of hypothalamic neurons (Cross and Green, 1959), (iii) glutaminergic activity in medulla-projecting neurons (Stocker et al., 2006), and finally (iv) increased activity of vasopressin-ergic spinal neurons (Antunes et al., 2006).

The complexity of the whole-animal osmoregulation system begins to emerge when one also considers the cardiovascular response to water consumption. Initially, this would be expected to increase plasma volume and decrease plasma osmolality. However, the response to water consumption in people is variable, depending on age and health status. In people with autonomic failure, consumption of moderate quantities of water evokes a substantial rise in BP of up to 100 mHg (Jordan et al., 1999; Cariga and Mathias, 2001; Lipp et al., 2005). This effect is absent in young, healthy human subjects (Jordan et al., 2000). The complex pattern of cardiovascular response to water consumption also includes increases in total peripheral resistance; presumably reflecting sympathetic vasomotor activity and a decrease in HR despite very little overall change in BP (Brown et al., 2005). This decrease in HR results from an increase in cardiac vagal drive (Routledge et al., 2002). Since these effects are seen with water, rather than oral consumption of isotonic saline (Lipp et al., 2005), plasma osmolality is clearly key. However, physiologically this may be an adaptation to rapidly redistribute plasma to the capacitance apparatus (Greenway and Lister, 1974), rather than simply initiating diuresis. This makes sense from an evolutionary context, since animals tend to preserve water and ions where possible (Share and Claybaugh, 1972). It appears that in healthy animals, blood volume increase is opposed by increasing (sympathetically driven) vascular tone, but resultant BP elevation is then limited by a vagal decrease in HR. This is more complex than a simple implementation of the baroreceptor reflex and whilst the site of such integrative control is not known, the hypothalamic PVN is well placed to contribute since it contains both sympathetic and vagal pre-autonomic neurons (Li et al., 2003). The PVN has been shown to be critical to the sympathetic nerve response to isotonic volume expansion (Haselton et al., 1994), but not the baroceptor reflex, which is centered in the medulla (Spyer, 1994).

Whilst there have been far fewer studies of “hypotonic responses” than there have been studies on responses to hypertonic exposure, there are some data available. For example, intra-carotid application of hypotonic solution decreases SNA and BP, but increases HR (Brooks et al., 2005). In the Brooks et al. (2005) study, these responses were only seen in water-deprived animals, however, the earlier study of Cross and Green (1959) showed suppression of action potential activity in the hypothalamus following intra-carotid hypotonic saline. In our own *ex vivo* “brain-slice” work, we found that a proportion of parvocellular neurons did respond to direct application of hypotonic solutions (Feetham et al., 2014). This would certainly have been expected for magnocellular neurons, which would switch off vasopressin release and thus increase diuresis, but was unexpected in parvocellular neurons. In our *ex vivo* work we established that TRPV4 was a critical element in osmosensing. This finding is consistent with the observations that TRPV4 is expressed in the PVN (Carreno et al., 2009) and that TRPV4^-/-^ KO mice are unable to detect hypo-osmolarity and respond with diuresis (Liedtke and Friedman, 2003; Mizuno et al., 2003).

In the current work we therefore investigated whether direct intra-cerebroventricular application of hypotonic saline to healthy CD1 mice modulated their cardiovascular parameters, and if so, whether this response was sensitive to the TRPV4 antagonist RN1734.

## Materials and Methods

### Immunohistochemistry

Immunohistochemistry was performed using a rabbit primary antibody for TRPV4 (1:300; Abcam, UK), and goat secondary antibody anti-rabbit CY3 (1:300; Abcam, UK). Blue DAPI dye was applied as a nuclear counter-stain, using VECTASHIELD mounting medium with DAPI (Vector laboratories, UK). Two types of negative control were used; one was omission of primary antibody, the second was the use of a specific TRPV4 blocking peptide (Abcam, UK). Blocking peptide was first incubated for 2 h with the primary antibody and then added to the slides along with the primary antibody throughout its incubation.

### Cannulation

Adult CD1 wild-type male mice (30–40 g) were anesthetized with a combination of urethane and α-chloralose (Sigma–Aldrich, UK), administered at an appropriate dose IP in saline. Urethane was used to minimize the effects on the cardiovascular system (Carruba et al., 1987). Following injection of the anesthetic, the mice were returned to their cage for several minutes until they lost consciousness. Body temperature was recorded immediately and continuously by rectal probe and maintained at 37 ± 0.5∘C by use of a heat lamp. Once loss of paw-withdrawal and eye-blink reflexes was achieved the trachea was intubated in order to maintain respiration. The carotid artery was cannulated with stretched PE25 tubing filled with heparinised saline. BP was recorded by a pressure transducer attached to the tubing and connected to a NeuroLog (Digitimer Ltd, Herefordshire, UK) BP amplifier. BP signals were digitized to PC with a CED Micro1401 (Cambridge Electronic Design, Cambridge, UK) using WinEDR at 5 kHz.

### Heart Rate Measurement

Heartbeats were annotated to the amplified AC coupled BP signal using Wabp from the PhysioNet suite of programs to give a HR read out (Goldberger et al., 2000). Briefly, the signal was analyzed at 1/10th sampling frequency (i.e., 500 Hz), and resampled to 125 Hz for optimal beat detection by Wabp. Annotated beats were then reverted to 10 times speed to give the actual HR.

### Intracerebroventricular Injections

For ICV injections the anesthetized mice were placed in a stereotaxic frame adapted for mice (Kopf instruments). Bregma was located according to the Paxinos and Franklin (2001) adult mouse stereotaxic atlas); a 2 mm craniotomy, 1 mm lateral, and 0.2 mm caudal to bregma allowed for drug or vehicle to be applied via 10 μL Hamilton syringe. All drugs were given in ACSF as the vehicle, and injected in a 1 μL volume gradually over a 30 s period. All injections were given into the lateral ventricle at the following coordinates; 0.2 mm caudal, 1 mm lateral, 3.2 mm vertical. The syringe was left at the injection site for 2 min and elevated to just above the injection site after this time, where it was kept in place for the duration of the recording. At the end of the procedure all animals were injected with 1% pontamine blue dye (Sigma–Aldrich, UK) at the same injection site using the same volume in order to confirm the correct location for the injection site. Post mortem, the brain removed and sliced to 300 μm on a Campden Instruments Ltd. 752 M Vibroslice to locate the injection site.

### Drug Injections

Drugs and vehicle controls used were; 1 μL isotonic/isotonic + DMSO ACSF, hypotonic ACSF (∼280 mOsmol) and RN1734 (Tocris, UK) in vehicle (ACSF; 100 nmol/kg).

### Statistical Analysis

Means are given ± SE (*n* = number of subjects). Hypothesis testing between two means was conducted with a *t*-test and where there were more than two groups a one way ANOVA was employed with Tukey’s multiple comparison adjustment. Statistical tests were conducted in Minitab (Minitab Ltd., Coventry, UK).

## Results

**FIGURE 1:**
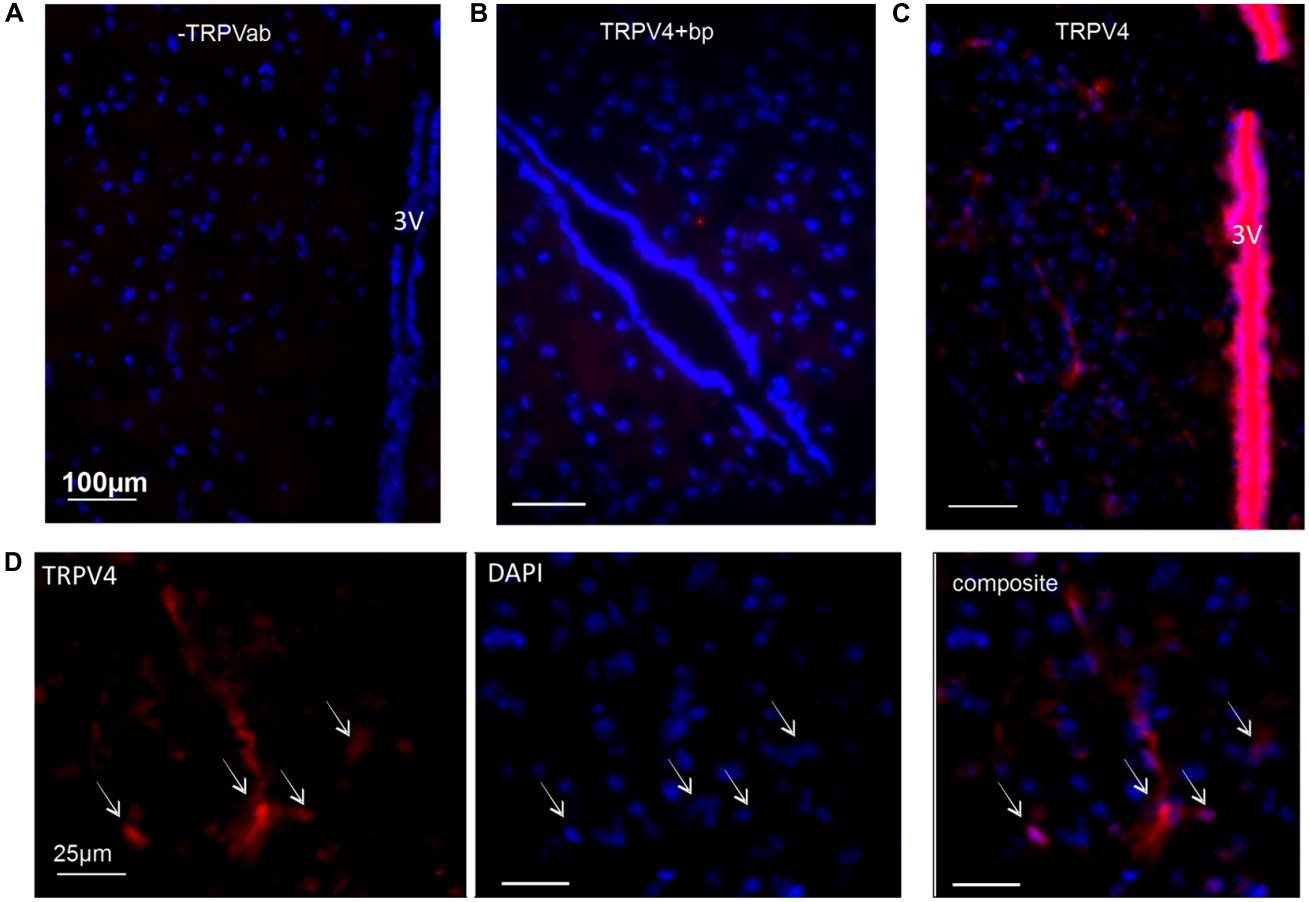
**Immunofluorescent identification of TRPV4 in the paraventricular nucleus**. PVN coronal slice labeled with antibody to TRPV4. **(A)** Negative control showing DAPI blue (a nuclear stain), with the absence of TRPV4 antibody staining (-TRPV4ab). Scale bar 100 μm and 3V indicates the third ventricle. **(B)** TRPV4 antibody applied together with a blocking peptide included (TRPV4 + bp). Red staining would indicate TRPV4 immunoreactivity, blue is DAPI nuclear staining. Scale bar 100 μm and 3V indicates the third ventricle.**(C)** Red staining indicates TRPV4 immunoreactivity, blue is DAPI nuclear staining. Scale bar 100 μm and 3V indicates the third ventricle. **(D)** High magnification images of the section seen in **(C)**. Red staining is TRPV4 and blue is DAPI nuclear staining; arrows indicate where overlap can be seen. Scale bar is 25 μm in each panel.

**FIGURE 2:**
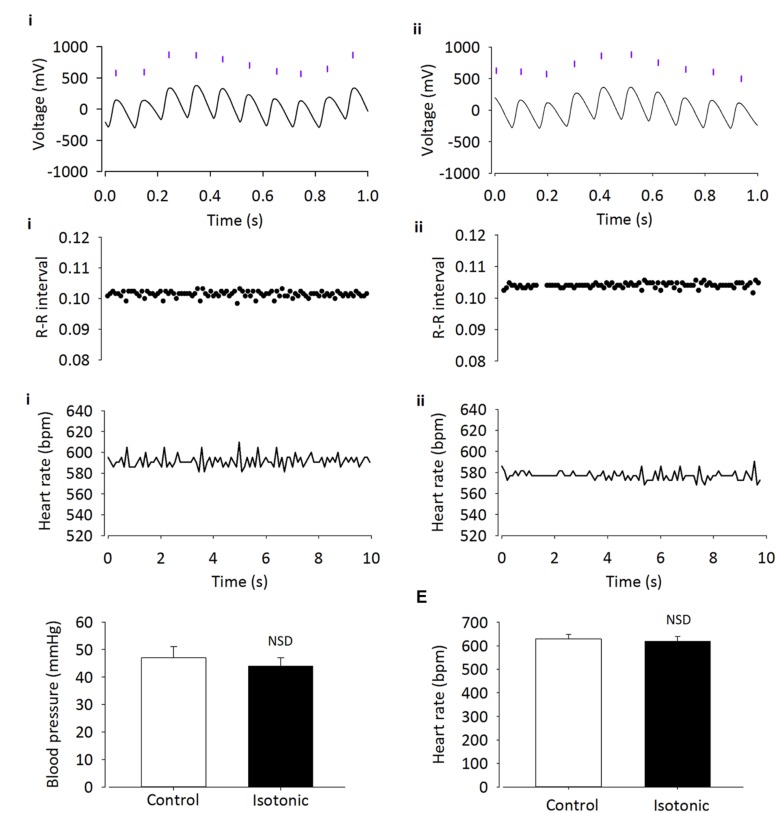
**Intracerebroventricular injection of isotonic ASCF has no effect on cardiovascular parameters**. Adult male CD1 mice were anesthetized with urethane–chloralose, and BP was recorded by cannulation of the carotid artery. **(A)** Raw BP trace with annotated beats (purple lines), before (i) and after (ii) injection of 300 mOsmol ACSF/DMSO vehicle. Annotated beats are used to derive R–R interval and HR. **(B)** Example R–R interval trace shows no difference before (i) and after (ii) ICV injection of isotonic ACSF. **(C)** Example HR trace shows no difference before (i) and after (ii) injection of isotonic ACSF. **(D)** Average BP and **(E)** HR do not change with injection of isotonic vehicle (*n* = 6; *p* > 0.05).

**FIGURE 3:**
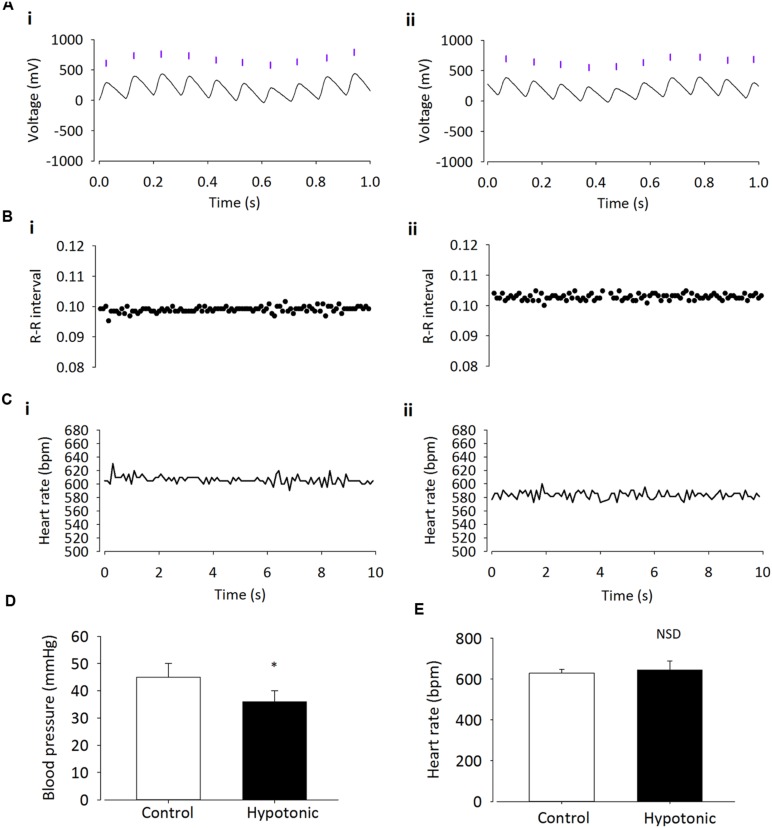
**Intracerebroventricular injection of hypotonic ASCF decreases BP but has no effect on HR**. BP significantly decreases after injection of hypotonic ACSF. **(A)** Raw BP trace with annotated beats (purple lines), before (i) and after (ii) injection. Annotated beats are used to derive R–R interval and HR. **(B)** Example R–R interval trace shows no difference before (i) and after (ii) ICV injection of hypotonic ACSF. **(C)** Example HR trace shows no difference before (i) and after (ii) injection of hypotonic ACSF. **(D)** Average BP is significantly reduced with injection of hypotonic ASCF (*n* = 6; ^∗^*p* < 0.01), but HR **(E)** remains unchanged (*p* > 0.05).

It has been suggested that TRPV4 may be responsible for volume control centrally (Bourque, 2008). Therefore we began by confirming a previous report (Carreno et al., 2009) of TRPV4 ion channel expression within the PVN using immunohistochemistry. We detected clear TRPV4 immunoreactivity within the parvocellular subnucleus of the PVN (**Figure 1**) and this was absent when a TRPV4 blocking peptide was included. Next, we investigated whether simple ICV injection had a confounding effect on cardiovascular parameters. In mice injected ICV with isotonic ACSF plus DMSO 0.01% (∼300 mOsmol) neither BP nor HR were significantly changed (**Figures 2 and 5**; *n* = 6; *p* > 0.05 by ANOVA using Tukey’s *post hoc* comparison). Next, ICV injections of 1 μL ∼270 mOsmol ACSF were given to investigate the central effects of hypo-osmolality on cardiovascular parameters. Hypotonic ACSF administered centrally resulted in a significant decrease in mean arterial pressure of -9 ± 2 mmHg (*n* = 6; *p* < 0.01 by ANOVA using Tukey’s *post hoc* comparison). No change in HR was observed (**Figures 2 and 5**; *n* = 6; *p* > 0.05 by ANOVA; **Figure 3**). Reduction in BP upon hypotonic injection was also significant compared to control (isotonic) ICV ACSF injection (**Figures 3 and 5**; -9 ± 2 mmHg vs. -2 ± 1 mmHg; *n* = 6; *p* < 0.01 by ANOVA with Tukey’s *post hoc* comparison).

**FIGURE 4:**
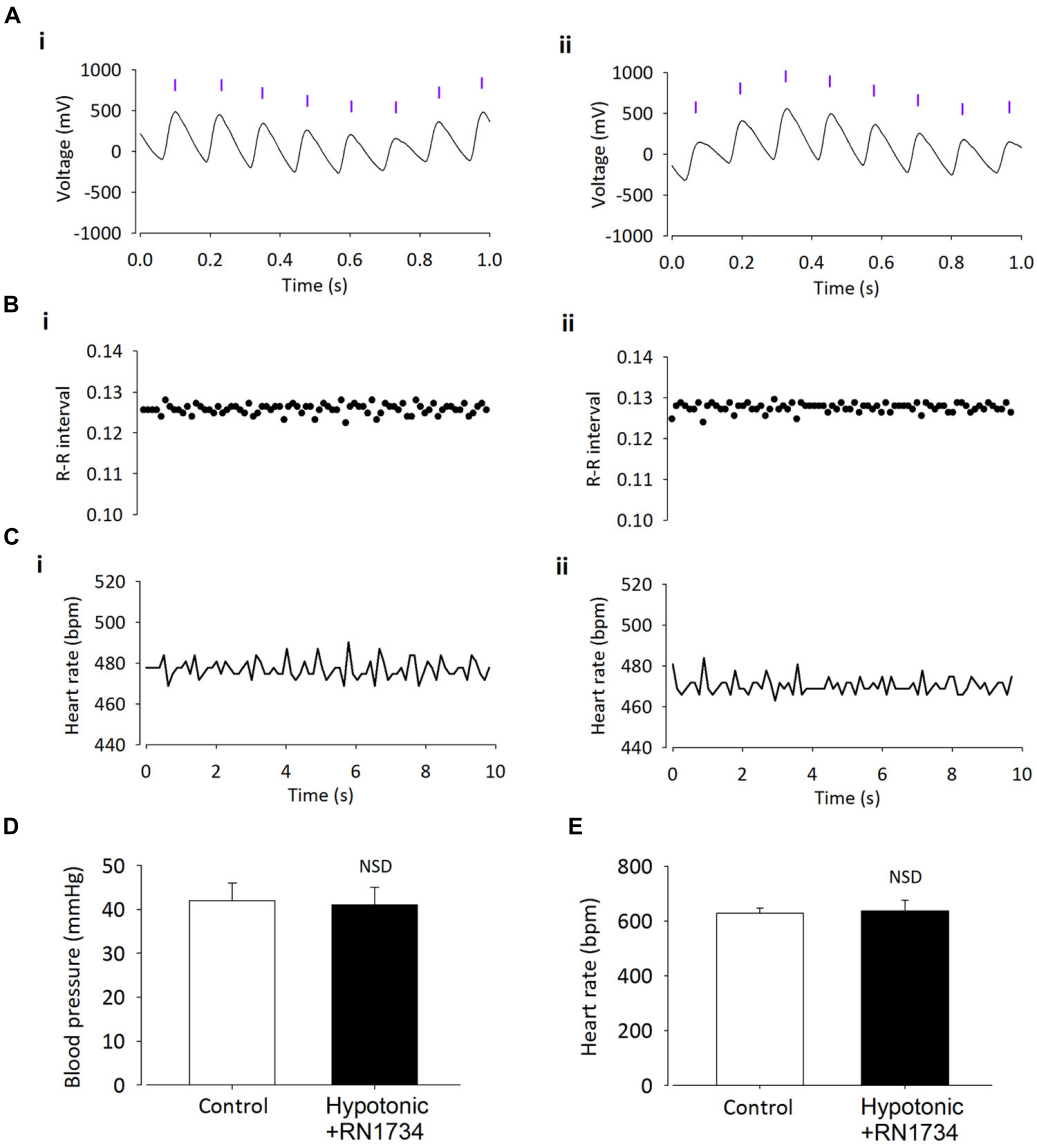
**Intracerebroventricular injection of the TRPV4 inhibitor RN1734 prevents the effect of hypotonic ACSF on BP. (A)** Raw BP trace with annotated beats (purple lines), before (i) and after (ii) injection. Annotated beats are used to derive R–R interval and HR. **(B)** Example R–R interval trace shows no difference before (i) and after (ii) ICV injection. **(C)** Example HR trace shows no difference before (i) and after (ii) ICV injection. **(D)** Average BP response to hypotonic ASCF is prevented by injection of RN1734 (*n* = 6; *p* > 0.05). **(E)** Average HR remains unchanged (*n* = 6; *p* > 0.05).

**FIGURE 5:**
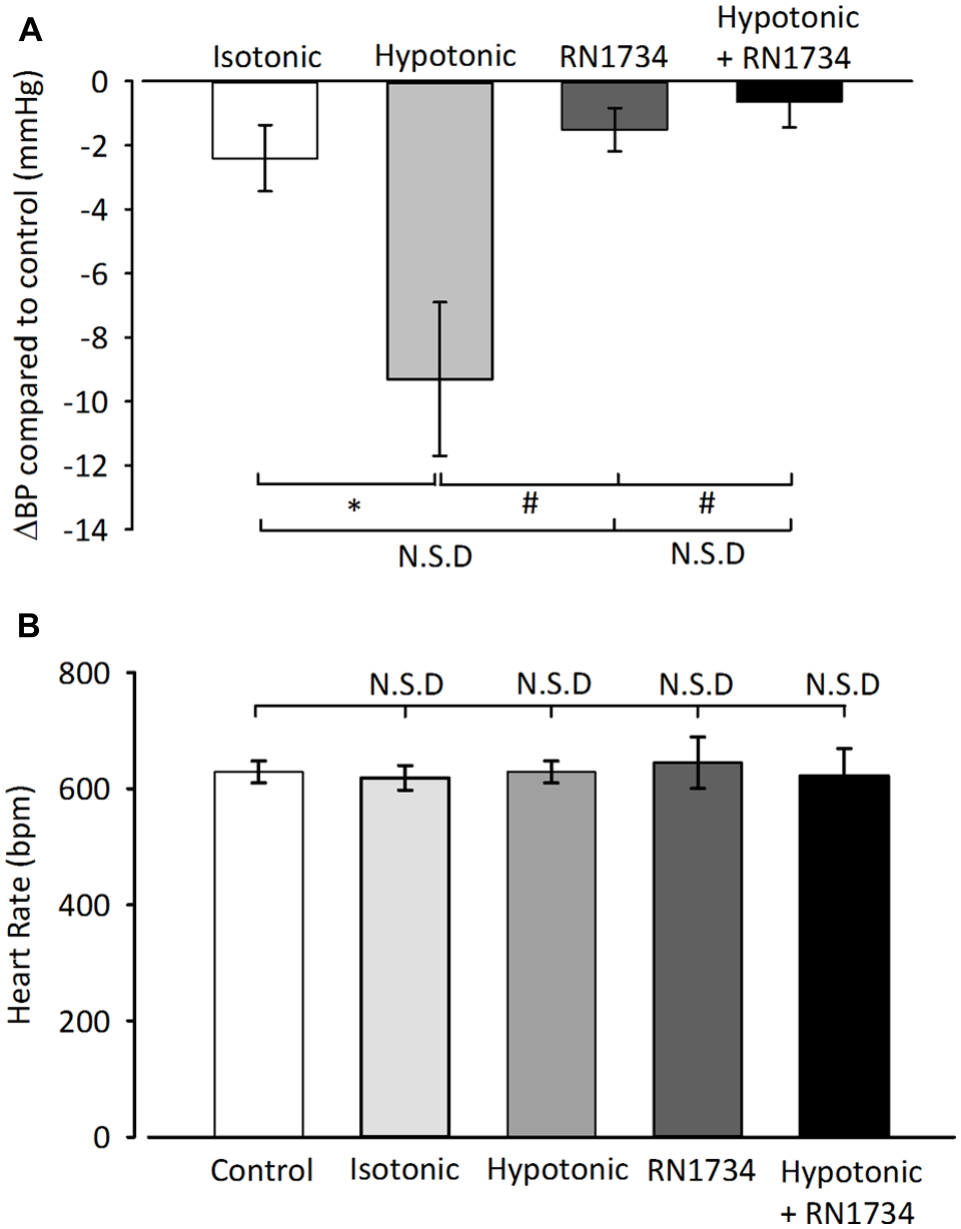
**Summary average changes in cardiovascular parameters from ICV injections. (A)** Average change in BP compared to control of several ICV injection treatments. No significant change was seen with vehicle (isotonic ACSF) or the TRPV4 inhibitor, RN1734 (100 nM/kg) alone vs. control (*n* = 6; *p* > 0.05). BP is significantly reduced in animals injected with hypotonic ACSF compared to those injected with vehicle (*n* = 6; ^∗^*p* < 0.01). ICV injections with RN1743 + hypotonic ACSF did not produce a significant BP change compared to vehicle injections (*n* = 6; *p* > 0.05), but BP was significantly reduced compared to hypotonic injections (*n* = 6; #*p* < 0.01). **(B)** Average HR did not change significantly between any of the conditions stated (*n* = 6; *p* > 0.05 by ANOVA).

Finally, we investigated whether the depressor action of hypotonic solution was dependent on TRPV4 channels. We tested this using 100 nM/kg of the selective TRPV4 inhibitor RN1734 (Vincent et al., 2009), along with hypotonic ASCF was injected ICV into anesthetized mice. Addition of the TRPV4 inhibitor prevented the reduction in mean arterial pressure observed with injection of hypotonic ACSF alone (**Figures 4 and 5**; *n* = 6; *p* > 0.05 by ANOVA using Tukey’s *post hoc* comparison). Again, no difference in HR was observed (**Figures 4 and 5**; *n* = 6; *p* > 0.05 by ANOVA using Tukey’s *post hoc* comparison).

## Discussion

In this study we identify a clear depressor action of euhydrated CD1 mice challenged with ICV hypotonic solution. This effect was prevented by treatment with the TRPV4 antagonist RN1734.

Several studies have indicated that the periventricular region of the brain is a key to detecting and responding to osmotic challenge (Toney et al., 2003; Stocker et al., 2007). Furthermore, our own work has demonstrated that, *in vitro*, PVN neurons can detect and respond to hypotonic solutions with a decrease in action potential firing (Feetham et al., 2014). *In vivo* the complex homeostatic response to PVN challenge with osmotic stimuli is likely to include both vasopressin release and activity of spinally projecting neurons; including interactions between these two pathways via dendritic–dendritic signaling (Stern, 2014).

Our previous report analyzed this coupling in fine detail and found that TRPV4 channels initially allow Ca^2+^ entry which, in turn, activates potassium channels and subsequently inhibits the firing of action potentials (Feetham et al., 2014). This effect is consistent with previous reports that the TRPV4 channel is known to be activated by osmolality changes (Liedtke et al., 2000) and has a role in volume control in other tissues (Becker et al., 2005; Guilak et al., 2010; Benfenati et al., 2011). Our combination of isolated neuron and brain slice experiments suggested that this is a direct effect within PVN parvocellular neurons rather than an indirect modulation of PVN projecting neurons. The latter, however, remains a possibility since spinally projecting PVN neurons receive functional inputs from a number of other hypothalamic nuclei (Cui et al., 2001; Stocker and Toney, 2005; Womack and Barrett-Jolley, 2007). This region of the PVN includes spinally projecting neurons which positively modulate the cardiovascular system (Coote, 2007) and so we hypothesized that such inhibition may result in a depressor action and reduction in HR. In the present study we find that whilst ICV hypotonic solutions do reduce BP, they have little effect on HR. Furthermore, the nature of this injection site means that it is not possible to know if the target neurons are in the PVN or some other periventricular site. It seems likely that this effect involves TRPV4 channels since these proteins are expressed in the PVN (**Figure 1** and see Carreno et al., 2009), and the TRPV4 inhibitor RN1734 (Vincent et al., 2009) abolished the effect. It has been shown previously that TRPV4^-/-^ mice have a diminished thirst response (Kinsman et al., 2014) although other work has shown that osmosensing in the periventricular area of the brain can also involve TRPV1 channels (Ciura and Bourque, 2006). Although TRPV1 involvement is a possibility, RN1734 is approximately 10× selective for TRPV4 over TRPV1 (Vincent et al., 2009) and our own *in vitro* work showed a TRPV4 dependent osmosensitivity of PVN neurons (Feetham et al., 2014). Future studies of this putative TRPV4 dependent mechanism could also use TRPV4^-/-^ mice. Absence of the response in such animals would provide further weight to the argument, although this approach too could provide its own complications.

It is important to note that it was necessary to conduct this study in anesthetized animals, which may have affected the response to hypotonicity. Anesthetics do affect the cardiovascular system; for example, by reducing resting BP. It is worth noting here that the recorded BP is quite low in this study compared to what one may expect in a conscious animal study, but is in line to that recorded previously in mice (Nunn et al., 2013).

These data support previous reports that changes in central osmolality result in the modulation of BP (Scrogin et al., 1999; Brooks et al., 2005). In previous studies altered HR has also been recorded upon osmotic change (Chen and Toney, 2001); however, our results show no statistically significant differences. This is not completely unexpected due to the baroreceptor reflex, which would be working to counteract the reduction in BP (Spyer, 1994).

Together, our current and previous data (Feetham et al., 2014) support the notion that there is a role for central TRPV4 channels in sensing osmolality changes and initiating changes in BP. This mechanism appears to operate within the PVN, but the exact phenotype of the active neurons is not known. Further investigation, perhaps in identified spinally projecting neurons, will be required to establish this. Potentially, pharmacological modulation of BP via TRPV4 or other RN1734 sensitive ion channels could be useful in the treatment of cardiovascular disease; however, the widespread distribution of TRPV4 ion channels could limit their practical usefulness. Therefore future studies will be aimed at identifying the receptor and neurotransmitter profile of PVN osmosensing neurons to determine if we can identify more specific therapeutic targets.

## Conflict of Interest Statement

The authors declare that the research was conducted in the absence of any commercial or financial relationships that could be construed as a potential conflict of interest.

## Abbreviations

ACSF: artificial cerebrospinal fluid
ACTH: adrenocorticotropic hormone
Ang II: angiotensin II
CRF: corticotrophin releasing factor
DMSO: dimethyl sulfoxide
GABA: γ-aminobutyric acid
ICV: intracerebroventricular
IML: intermediolateralis
IP: intraperitoneal
K_Ca_ Ca^2+^: activated K^+^ channel
KO: knock-out
MPO: Medial Preoptic Area
NMDA: *N*-methyl-D-aspartate receptor
NO: nitric oxide
NOS: nitric oxide synthase
PVN: paraventricular nucleus
RSNA: renal sympathetic nervous activity
SCN: suprachiasmatic nucleus
SFO: subfornical organ
SNA: sympathetic nervous activity
SNS: sympathetic nervous system
TRP: transient receptor potential
TRPV: transient receptor potential vanilloid
